# Effects of Dietary Baicalin on Aflatoxin B1-Induced Growth Performance and Liver Health in Ducklings

**DOI:** 10.3390/ani15162325

**Published:** 2025-08-08

**Authors:** Qirong Lu, Xue Zhang, Jie Zhang, Xinyue Wang, Defeng Wen, Pu Guo, Jianglin Xiong, Yinsheng Qiu

**Affiliations:** 1 Hubei Key Laboratory of Animal Nutrition and Feed Science, School of Animal Science and Nutritional Engineering, Wuhan Polytechnic University, Wuhan 430023, China; qirongluvet@whpu.edu.cn (Q.L.);; 2 Wuhan Engineering and Technology Research Center of Animal Disease-Resistant Nutrition, School of Animal Science and Nutritional Engineering, Wuhan Polytechnic University, Wuhan 430023, China; 3 National Reference Laboratory of Veterinary Drug Residues (HZAU) and MAO Key Laboratory for Detection of Veterinary Drug Residues, Huazhong Agricultural University, Wuhan 430070, China

**Keywords:** aflatoxin B1, baicalin, ducklings, liver injury, growth performance

## Abstract

Aflatoxin B1 (AFB1) is a highly toxic secondary metabolite produced by *Aspergillus* species. Its extensive contamination of animal feed and human food poses significant health hazards to livestock and humans, with hepatotoxicity being a primary concern. This study investigated the protective effect of baicalin on AFB1-induced liver injury in ducklings. In the first experiment, ducklings were administered AFB1 at doses of 0, 6, 12, or 24 μg/kg body weight/day for 7 days to identify the optimal concentration for establishing a model of AFB1-induced growth performance and liver injury. Administration of AFB1, particularly at the higher doses (12 and 24 μg/kg body weight/day), significantly reduced growth performance and induced structural and functional liver injury (*p* < 0.05). In a second experiment, ducklings were administered AFB1 (12 μg/kg body weight/day) with or without baicalin (25–100 mg/kg body weight/day) for 7 days. Dietary baicalin significantly increased the serum albumin level; reduced the serum alanine aminotransferase, aspartate aminotransferase, and gamma-glutamyl transferase levels; improved growth performance; and ameliorated structural and functional liver injury in ducklings exposed to AFB1 (*p* < 0.05). The results indicate that dietary baicalin alleviates AFB1-induced liver injury and growth performance decline in ducklings. Hence, baicalin could serve as a potential feed additive to reduce the harmful effects of AFB1 in the poultry breeding industry.

## Simple Summary

Aflatoxin B1 (AFB1), a toxic compound commonly found in contaminated animal feed, can severely damage liver health and reduce growth performance in ducklings. This study explored whether baicalin, a natural flavonoid from *Scutellaria baicalensis*, could protect against such damage. The results showed that dietary baicalin effectively improved liver function, reduced harmful enzyme levels, and boosted overall performance in AFB1-exposed ducklings. These findings suggest baicalin may serve as a beneficial feed additive in poultry production to combat aflatoxin toxicity.

## 1. Introduction

Aflatoxin B1 (AFB1) is a secondary metabolite produced by *Aspergillus* species [1,2]. As the most toxic aflatoxin subtype in feed, AFB1 seriously affects the health of livestock and poultry, and causes huge economic losses to livestock and poultry [3]. In one study, the authors analyzed the concentration of AFB1 in 74,821 feeds and feed raw materials collected from 100 countries from 2008 to 2017. The analysis revealed the global presence of AFB1 in 74,821 feeds and feed raw materials [4]. AFB1-contaminated feed consumed by livestock and poultry can cause liver injury, as the liver is the target organ for toxin activation, metabolism, and elimination [5]. AFB1 in contaminated feed consumed by livestock and poultry accumulates in the liver and can be transmitted to humans through the food chain, posing a serious threat to animals and humans [6,7]. A previous study has reported that feeding 1-day-old ducks with a basal diet supplemented with 90 μg/kg AFB1 for 4 weeks resulted in significant body weight loss and severe hepatotoxicity [8]. Another study has reported that feeding nursery pigs continuously with 0.5 mg/kg AFB1 in the basal diet for 20 days decreased the growth rate and feed intake, accompanied by an increase in the levels of serum aspartate aminotransferase (AST) and alanine aminotransferase (ALT) [9]. Additionally, a study has reported that dietary consumption of 0.1 mg/kg AFB1 for 21 days significantly decreased the growth performance and hepatic function of broilers [10]. Furthermore, research has shown that female mutton sheep consuming a diet supplemented with 75 μg AFB1/kg dry matter for 30 days exhibited significant reductions in average daily feed intake (ADFI) and significant increases in serum ALT and AST levels [11]. Based on the aforementioned studies, dietary AFB1 exposure significantly impairs growth performance and liver function in livestock and poultry. Consequently, strategies to determine and mitigate the detrimental AFB1-induced effects in livestock and poultry have become a major focus of contemporary research [12,13,14,15].

Baicalin, a bioactive flavonoid compound extracted from *Scutellaria baicalensis*, has garnered considerable research attention due to its broad spectrum of pharmacological properties, including antimicrobial, anti-apoptotic, anti-inflammatory, antioxidant, and hepatoprotective activities [16,17,18]. In a model of duck liver injury induced by duck hepatitis A virus type 1, baicalin (3 mg/kg body weight) significantly increased the survival rate of ducklings and reduced hepatic mitochondrial dysfunction [19]. In a chicken liver injury model induced by zearalenone, baicalin (20, 40, or 80 mg/kg body weight) significantly reduced the serum levels of AST and ALT and inhibited the inflammatory response, apoptosis, and oxidative stress in the liver [20]. Moreover, in in vitro models, baicalin has been shown to alleviate hepatocyte injury caused by AFB1 via apoptosis and ferroptosis [21,22]. Therefore, baicalin supplementation has the potential to protect the liver from AFB1-related injury, but its utility as a feed additive for ducklings warrants in vivo validation in ducklings.

This study aimed to establish an experimental model of AFB1-induced liver injury in ducklings and to evaluate the hepatoprotective efficacy of dietary baicalin supplementation against this mycotoxin-mediated injury. The findings establish a theoretical foundation for the potential application of dietary baicalin supplementation to mitigate AFB1-induced health impairments in livestock and poultry.

## 2. Materials and Methods

### 2.1. Chemicals

Baicalin (CAS No.: 21967-41-9) was obtained from Chengdu Biopurify Phytochemicals Ltd. (Chengdu, China). AFB1 (CAS No.: 1162-65-8) was obtained from Qingdao Pribolab Biological Engineering Co., Ltd. (Qingdao, China).

### 2.2. Animal Experiment Design

In both study parts, one-day-old Liangping ducklings were purchased from the Changfeng Hatchery (Xiaonan District, Xiaogan City, China). One week prior to the experiment, the duck house and cages were thoroughly cleaned and rinsed. Subsequently, the duck house was sealed and fumigated with potassium permanganate and formaldehyde for disinfection. Following a 3-day fumigation period, the house was ventilated for 4 days. To ensure duckling comfort and minimize stress, the heating system maintained an ambient temperature of 34 °C for ducklings aged 1 to 3 days. The temperature was then gradually reduced to reach the target ambient temperature required for ducklings aged 4 to 14 days. Consistent nutritional conditions were maintained, with all ducklings across treatment groups receiving the identical basal diet. All ducklings were fed the same basic diet from days 1 to 3 of age.

In the first part of the study, 20 one-day-old Liangping ducklings were used. Beginning on day 4, the 20 ducklings were randomly divided into four groups (*n* = 5/group): one control group and three AFB1 challenge groups, specifically a low dose at 6 μg/kg body weight/day, a medium dose at 12 μg/kg body weight/day, and a high dose at 24 μg/kg body weight/day. Beginning on day 4, the ducklings were administered vehicle (control group) or AFB1 once a day for 7 consecutive days. All ducklings could freely eat and drink. After each feeding, the feeding, drinking, and activity of the ducklings were carefully observed, and the feed intake was accurately recorded. The dose of AFB1 were chosen based on previous studies that indicated exposure to 10–40 μg/kg AFB1 can significantly induce liver injury and affect the growth performance of ducklings [23,24], and the China national feed hygiene standard GB 13078-2017 stipulates that the maximum permissible concentration of AFB1 in feed for meat ducks and laying ducks is 15 μg/kg [25]. The animal experiments were approved by the Animal Ethics Committee of Wuhan Polytechnic University (permit number WPU202404003).

The medium AFB1 dose (12 μg/kg body weight/day) caused severe liver injury in ducklings, so this dose was selected for the second part of the study. Sixty 1-day-old Liangping ducklings were used. On day 4, the ducklings were randomly divided into six groups (*n* = 10/group): the control group, the AFB1 group (12 μg/kg body weight/day AFB1), the AFB1 + low-dose baicalin group (12 μg/kg body weight/day AFB1 + 25 mg/kg body weight/day baicalin), the AFB1 + medium-dose baicalin group (12 μg/kg body weight/day AFB1 + 50 mg/kg body weight/day baicalin), and the AFB1 + high-dose baicalin group (12 μg/kg body weight/day AFB1 + 100 mg/kg body weight/day baicalin). Baicalin was administered daily via oral gavage from day 4 until trial termination. Beginning on day 7, the ducklings in the AFB1 group received the AFB1 solution once a day for 7 consecutive days via oral gavage. The dose of baicalin was chosen based on previous studies that the hepatoprotective effects of baicalin against various toxins have utilized doses within the range of 25–200 mg/kg body weight [26,27,28,29]. All ducklings could freely feed and drink. After each feeding, the feeding, drinking, and activity behaviors of the ducklings were carefully observed, and the feed intake was accurately recorded. The animal experiments were approved by the Animal Ethics Committee of Wuhan Polytechnic University (permit number WPU202406002).

### 2.3. Serum Biochemical Assay

For the biochemical parameters, heparinized blood samples were centrifuged at 3500 rpm for 10 min to collect serum. An automatic blood biochemical analyzer (HITACHI 7100, Hitachi, Tokyo, Japan) was used to determine the serum albumin (ALB), ALT, AST, and gamma-glutamyl transferase (GGT) levels.

### 2.4. Histopathological Observation

After collecting liver samples from the ducklings, they were fixed in 4% paraformaldehyde (Servicebio, Wuhan, China). After embedding in paraffin, the samples were sectioned and stained with hematoxylin and eosin (H&E) by Wuhan Pigeonbio Technology Co., Ltd. (Wuhan, China). The histopathological changes in liver tissues were observed using a microscope and an imaging system (Nikon, Tokyo, Japan).

### 2.5. Statistical Analysis

Statistical analysis was performed using SPSS Statistics 19.0 for Windows (IBM Corp., Armonk, NY, USA). The data are presented as the mean and standard error of the mean. One-way analysis of variance was used to analyze the data, followed by Duncan’s post hoc analysis, with *p* < 0.05 indicating a significant difference between groups and *p* < 0.01 indicating a highly significant difference.

## 3. Results

### 3.1. Growth Performance of Ducklings Exposed to Different Doses of AFB1

Table 1 shows the effects of different AFB1 doses on the growth performance of ducklings. Compared with the control group, the average daily gain (ADG) of ducklings in the 12 and 24 μg/kg body weight/day groups was significantly reduced (*p* < 0.05), and the ADFI showed a decreasing trend, but there was no significant difference between the groups (*p* > 0.05). Meanwhile, the feed-to-gain ratio (FCR) was not significantly different between the groups (*p* > 0.05). Intake of 12 and 24 μg/kg body weight/day AFB1 had a significant inhibitory effect on the growth of ducklings, while intake of 6 μg/kg body weight/day AFB1 did not.

### 3.2. The Effect of Different Doses of AFB1 on Liver Histopathology of Ducklings

**Figure 1:**
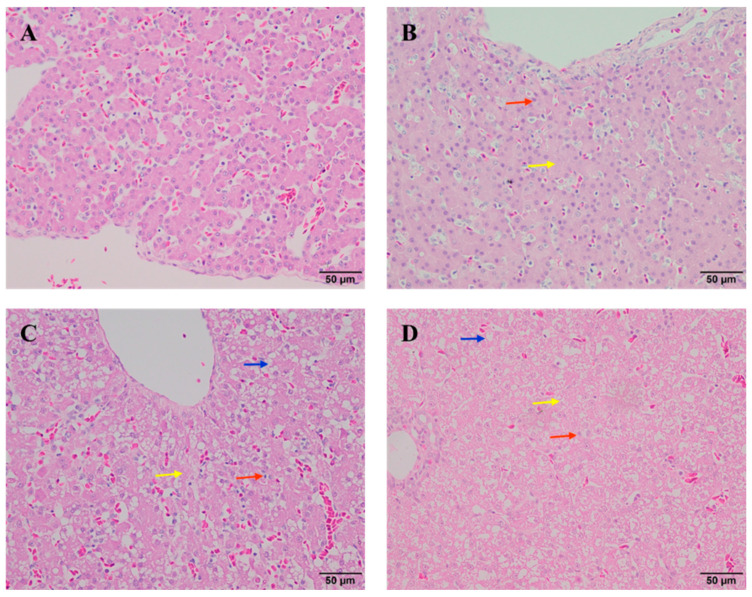
Histopathological changes in liver in ducklings induced by different doses of aflatoxin B1 (AFB1) (haematoxylin and eosin staining, 400 × magnification). (**A**) Control group; (**B**) 6 μg/kg body weight/day AFB1 group; (**C**) 12 μg/kg body weight/day AFB1 group; (**D**) 24 μg/kg body weight/day AFB1 group. The yellow arrows in the figure indicate hepatocyte granular degeneration. The blue arrow shows hepatocyte steatosis. The red arrow shows liver cell necrosis.

Figure 1 shows images of H&E-stained liver samples from the ducklings. In the control group (Figure 1A), the liver tissue structure was normal, the hepatocytes were arranged neatly without obvious cell degeneration, the liver lobules were clearly visible and structurally intact, the hepatocytes centered on the portal vein were arranged radially, and the nucleoli were large and clear. Compared with the control group, there was granular degeneration of hepatocytes or steatosis in the 6 μg/kg body weight/day AFB1 group (Figure 1B). In the 12 μg/kg body weight/day AFB1 group (Figure 1C), the liver showed a disordered arrangement of hepatocyte cords, most hepatocytes had undergone granular degeneration and steatosis, and there was focal hepatocyte necrosis. In the 24 μg/kg body weight/day AFB1 group (Figure 1D), most hepatocytes had undergone granular degeneration or steatosis, and many hepatocytes had undergone necrosis. Overall, 6 μg/kg body weight/day AFB1 caused less intense liver injury in ducklings compared with the two higher concentrations.

### 3.3. The Effect of Baicalin on Growth Performance of AFB1-Exposed Ducklings

Table 2 presents the effects of baicalin on the growth performance of AFB1-exposed ducklings. Compared with the control group, the ADG was significantly decreased in the AFB1 group (*p* < 0.05). There was no significant difference in the ADFI and FCR between control and AFB1 groups (*p* > 0.05). Compared with the AFB1 group, the ADG was significantly increased in the AFB1 + 100 mg/kg body weight/day baicalin group (*p* < 0.05). Compared with the AFB1 group, the FCR was significantly reduced in the AFB1 + 100 mg/kg body weight/day baicalin group (*p* < 0.05). Overall, AFB1 challenge significantly reduced ADG, and the addition of 100 mg/kg body weight/day baicalin after this challenge significantly restored growth performance.

### 3.4. The Effect of Baicalin on Serum Biochemical Parameters of AFB1-Exposed Ducklings

The serum levels of ALB, ALT, AST, and GGT were used as indicators of liver injury (Table 3). Compared with the control group, the AFB1 group exhibited a significant decrease in serum ALB (*p* < 0.05) and significant increases in serum AST, ALT, and GGT (*p* < 0.05). Compared with the AFB1 group, all baicalin treatment groups (25, 50, and 100 mg/kg body weight/day) significantly increased serum ALB (*p* < 0.05) and significantly decreased serum ALT and AST (*p* < 0.05). Notably, the higher dose of baicalin (100 mg/kg body weight/day) additionally induced a significant decrease in serum GGT (*p* < 0.05). These data indicate that baicalin protected ducklings against AFB1-induced liver injury.

### 3.5. The Effect of Baicalin on Liver Histopathological Changes in AFB1-Exposed Ducklings

**Figure 2:**
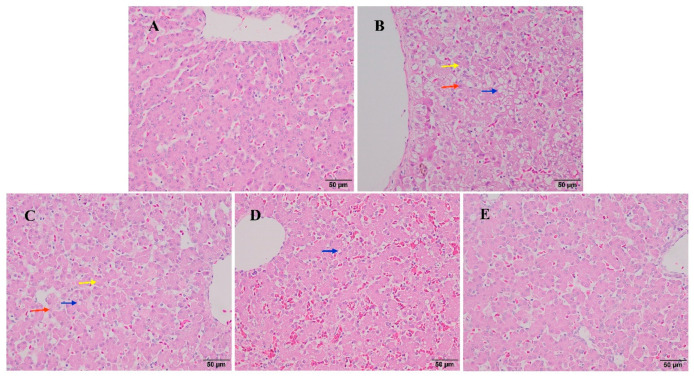
The effect of baicalin on liver histopathological changes in ducklings exposed to AFB1 (haematoxylin and eosin staining, 400× magnification). (**A**) control group; (**B**) 12 μg/kg body weight/day AFB1; (**C**) 12 μg/kg body weight/day AFB1 + 25 mg/kg body weight/day baicalin; (**D**) 12 μg/kg body weight/day AFB1 + 50 mg/kg body weight/day baicalin; (**E**) 12 μg/kg body weight/day AFB1 + 100 mg/kg body weight/day baicalin. The yellow arrow shows hepatocyte granular degeneration. The blue arrow shows hepatocyte steatosis. The red arrow shows liver cell necrosis.

Figure 2 shows the effect of baicalin on the histopathological changes in the liver of AFB1-exposed ducklings. In the control group (Figure 2A), the liver tissue showed a normal structure, with neatly arranged hepatocytes without obvious cell degeneration, clearly visible and structurally intact liver lobules, the hepatocytes centered on the portal vein were arranged radially, and large and clear nucleoli. The AFB1 group (Figure 2B) presented a disordered arrangement of hepatocyte cords, and most hepatocytes had undergone granular degeneration and steatosis, with focal hepatocyte necrosis. Compared with the AFB1 group, the AFB1 + 25 mg/kg body weight/day baicalin group (Figure 2C) showed no obvious changes in the liver structure, although most hepatocytes had undergone granular degeneration or steatosis, and a few hepatocytes had undergone necrosis. In the AFB1 + 50 mg/kg body weight/day baicalin group (Figure 2D), vacuolar degeneration and steatosis of hepatocytes were reduced, and there was less extensive hepatocyte granular degeneration or steatosis. There was significantly improved hepatocyte degeneration in the AFB1 + 100 mg/kg body weight/day baicalin group (Figure 2E), with no significant difference in hepatocytes compared with the control group. Taken together, administration of 50 and 100 mg/kg body weight/day baicalin alleviated AFB1-induced liver injury in ducklings, with the higher dose producing better results.

## 4. Discussion

AFB1 is a very common mycotoxin in feed; it contaminates a wide range of feed and feed raw materials and thus poses a serious threat to the health of livestock and poultry [3,30,31]. Exposure to AFB1 can seriously affect the growth performance of livestock and poultry and lead to hepatoxicity [32,33,34]. Natural products and dietary supplements have been shown to alleviate the injury caused by AFB1 exposure [35,36,37]. It is of great clinical significance to study the growth performance and hepatotoxicity caused by AFB1 exposure in ducklings and the regulatory effects of natural products. Thus, we investigated the protective effect of baicalin against AFB1-induced liver injury and growth performance decline in ducklings. We found that dietary baicalin significantly improved the growth performance and liver histopathological changes in ducklings exposed to AFB1.

AFB1 is a prevalent contaminant in agricultural products, presenting significant risks to the growth performance of livestock and poultry, including ducks [24]. In a previous study, challenge with 20 μg/kg body weight AFB1 significantly decreased the body weight and ADG of male Pekin ducklings [24]. In another study, compared with the control group, 7-day-old Cherry Valley ducklings fed 0.1 mg AFB1/kg exhibited a reduction in the final body weight and the ADG, and a poor FCR during a 42-day trial [38]. One-day-old Cherry Valley ducklings challenged with 120.02 μg/kg AFB1 presented a significant reduction in the ADG and ADFI and an increase in mortality during days 0–14, while 153.12 μg/kg AFB1 challenge did not affect the FCR during days 15–35 [39]. Consistent with these results, we found that the ADG of ducklings exposed to 12 or 24 μg/kg body weight/day AFB1 for 7 days decreased significantly and dose dependently, and the ADFI showed a trend for a decrease. The findings from previous studies and our study imply that feeding ducklings AFB1-contaminated feed can affect growth performance, so it is critical to seek interventions to reduce the impact of AFB1 on growth performance.

Baicalin is a flavonoid extracted from *S. baicalensis* with a variety of pharmacological effects, including antitumor, antiviral, antibacterial, neuroprotective, and especially hepatoprotective [16,40,41,42]. Meanwhile, the extract of *S. baicalensis* (containing ≥85% baicalin) demonstrates potential as a feed additive to enhance livestock and poultry growth performance [43]. Based on this, it is anticipated that baicalin can be developed as a feed additive for application in animal production. We found that 100 mg/kg body weight/day baicalin significantly decreased the FCR and increased the ADG in AFB1-exposed ducklings. Abnormal serum levels of ALB, ALT, AST, and GGT have been reported as indicators of hepatotoxicity [44,45]. After treatment with baicalin, the ALT and AST levels in ducklings infected with duck hepatitis A virus type 1 were significantly reduced [19,46], demonstrating the hepatoprotective effect of baicalin. In rats, baicalin attenuated the AFB1-induced increase in serum ALT and AST and hepatocyte injury [22]. In addition, baicalin treatment significantly reduced the levels of ALT, AST, and GGT in a rat model of ulcerative colitis, thereby alleviating liver injury [47]. Consistently, we found that baicalin significantly reduced serum AST, ALT, and GGT and increased serum ALB in ducklings after AFB1 challenge. These biochemical changes were accompanied by a reduction in AFB1-induced liver injury.

The liver is the most important detoxification organ for AFB1, and it is also the target organ of this toxin [48,49]. Liver histopathological changes are the key indicators of AFB1-mediated toxicity [50,51]. A previous study reported that supplementing the basal diet with 50 μg/kg AFB1 caused severe liver injury in ducks, including vacuolar degeneration, inflammatory cell infiltration, cell necrosis, and swelling [44]. In another study, H&E staining of the liver of ducklings exposed to 0.1 mg/kg AFB1 showed a disordered hepatic cord—it sometimes even disappeared—and the hepatocytes appeared to have undergone vacuolization and steatosis [52]. One-day-old Sichuan Sheldrakes exposed to 100 μg/kg AFB1 showed obvious destruction of liver lobule structure, obvious vacuolar degeneration of hepatocytes, distorted cell structure, nuclear pyknosis, obvious inflammatory cell infiltration, and partial cell degeneration and necrosis [53]. Consistently, the AFB1-challenged ducklings in the present study showed a disordered arrangement of hepatocyte cords, granular degeneration and steatosis of most hepatocytes, and focal hepatocyte necrosis. Previous studies indicated that AFB1 exposure induces multifaceted liver injury in ducklings, including oxidative stress via suppression of the nuclear factor erythroid 2-related factor 2-heme oxygenase 1 pathway [7,44,54], inflammatory injury mediated by nod-like receptor protein 3 inflammasome activation [5,55], and apoptosis triggered by pro-apoptotic signaling [33,54]. The present study showed that baicalin significantly protected against AFB1-induced histopathological changes in the liver. However, the molecular mechanism underlying this protective effect, such as potential roles of antioxidative, anti-inflammatory, and anti-apoptotic pathways, requires further elucidation.

However, this study has several limitations. First, the molecular mechanisms by which baicalin alleviates AFB1-induced liver injury require comprehensive elucidation in both in vivo models (specifically ducklings) and in vitro systems. Second, the protective efficacy of baicalin against AFB1-induced liver injury warrants verification across diverse duck breeds and other poultry species. Third, the impact of AFB1–baicalin interactions on gut microbiota dysbiosis, a critical modulator of hepatotoxicity, remains unexplored. Fourth, the long-term protective efficacy of baicalin remains unassessed.

## 5. Conclusions

We found that dietary baicalin reduced AFB1-induced liver injury, denoted by a reduction in serum biochemical indicators of liver injury and liver pathological changes. These findings indicate that baicalin is an effective feed additive that can potentially protect ducklings from AFB1-induced liver injury by improving growth performance and reducing liver injury. Its use for the safe production of livestock and poultry could reduce the economic losses caused by AFB1 contamination.

## Figures and Tables

**Table 1:** The effect of aflatoxin B1 (AFB1) intake dose on growth indicators of ducklings. In each row, different lowercase superscript letters indicate a significant difference (*p* < 0.05), while the same lowercase superscript levels indicate no significant difference (*p* > 0.05). ADG: average daily gain; ADFI: average daily feed intake; FCR: feed-to-gain ratio. “/” indicates there was no separate cage feeding, and there was only one data point for ADFI and FCR for the group.

Indicators	Control	AFB1 (μg/kg Body Weight/Day)			SEM	*p* Value
		6	12	24		
ADG (g/day)	28.64 ^a^	27.57 ^a^	24.29 ^b^	21.74 ^b^	0.779	0.001
ADFI (g/day)	58.66	56.06	52.87	50.77	/	/
FCR	1.98	2.03	2.17	2.31	/	/

**Table 2:** The effect of baicalin on growth performance of aflatoxin B1 (AFB1)-challenged ducklings. AFB1: 12 μg/kg body weight/day AFB1; 25BA: 12 μg/kg body weight/day AFB1 + 25 mg/kg body weight/day baicalin; 50BA: 12 μg/kg body weight/day AFB1 + 50 mg/kg body weight/day baicalin; 100BA: 12 μg/kg body weight/day AFB1 + 100 mg/kg body weight/day baicalin. ADG: average daily gain; ADFI: average daily feed intake; FCR: feed-to-gain ratio.

Indicators	Control (A)	AFB1 (B)	25BA (C)	50BA (D)	100BA (E)	SEM	*p* Value			
							A vs. B	C vs. B	D vs. B	E vs. B
ADG (g/day)	26.48	23.10	24.16	26.68	27.84	0.531	0.005	0.942	0.162	0.015
ADFI (g/day)	51.60	49.83	44.99	48.14	48.80	1.118	0.572	0.474	0.969	0.995
FCR	1.96	2.13	1.86	1.84	1.75	0.050	0.074	0.173	0.141	0.035

**Table 3:** The effect of baicalin on serum biochemical parameters of aflatoxin B1 (AFB1) exposed ducklings. AFB1: 12 μg/kg body weight/day AFB1; 25BA: 12 μg/kg body weight/day AFB1 + 25 mg/kg body weight/day baicalin; 50BA: 12 μg/kg body weight/day AFB1 + 50 mg/kg body weight/day baicalin; 100BA: 12 μg/kg body weight/day AFB1 + 100 mg/kg body weight/day baicalin. ALB: albumin; ALT: alanine aminotransferase; AST: aspartate aminotransferase; GGT: gamma-glutamyl transferase.

Indicators	Control (A)	AFB1 (B)	25BA (C)	50BA (D)	100BA (E)	SEM	*p* Value			
							A vs. B	C vs. B	D vs. B	E vs. B
ALB (g/L)	11.76	10.43	11.77	11.83	12.24	0.214	0.040	0.027	0.030	0.005
AST (U/L)	22.00	35.13	22.60	20.50	21.00	2.078	0.013	0.010	0.002	0.005
ALT (U/L)	49.25	63.00	44.30	49.86	44.70	2.257	0.015	0.002	0.026	0.003
GGT (U/L)	4.40	7.10	5.00	5.33	4.70	0.193	0.020	0.063	0.160	0.027

## Data Availability

The data presented in this study are available in the article.

## Abbreviations

The following abbreviations are used in this manuscript:

ADFI	Average daily feed intake
ADG	Average daily gain
AFB1	Aflatoxin B1
ALB	Albumin
ALT	Alanine aminotransferase
AST	Aspartate aminotransferase
FCR	Feed-to-gain ratio
GGT	Gamma-glutamyltransferase
H&E	Hematoxylin and eosin
